# Analysis of latent profiles and influencing factors of artificial intelligence anxiety among newly recruited nurses: a cross-sectional study

**DOI:** 10.3389/fpubh.2026.1903509

**Published:** 2026-07-14

**Authors:** Xinru Ma, Huan Wang

**Affiliations:** School of Nursing, Jinzhou Medical University, Jinzhou, China

**Keywords:** AI anxiety, artificial intelligence, latent profile analysis, newly recruited nurses, nurses

## Abstract

**Objective:**

To investigate the current status of artificial intelligence (AI) anxiety among newly recruited nurses, explore its latent categories and characteristics, and analyze related influencing factors, thereby providing a scientific basis for promoting the acceptance of AI technology among newly recruited nurses and enhancing the nursing workforce’s adaptability to AI applications.

**Methods:**

Convenience sampling was used to select 715 newly recruited nurses from four Grade A tertiary hospitals in Liaoning, Shandong, and Jilin, China. Data were collected using a demographic questionnaire, the Artificial Intelligence Anxiety Scale, the Attitude Scale toward the Use of Artificial Intelligence Technology in Nursing, and the General Self-Efficacy Scale. Latent profile analysis was conducted using Mplus 8.3 software to explore the categories and characteristics of AI anxiety among newly recruited nurses, and univariate analysis and multivariate logistic regression analysis were performed using SPSS 26.0 software to investigate the influencing factors of different categories.

**Results:**

AI anxiety among newly recruited nurses was classified into three categories: low anxiety-technology acceptance (47.6%), moderate anxiety-ambivalent watch and wait (37.5%), and high anxiety-technology rejection (15.0%). Educational level, income level, experience with AI training, proficiency in AI technology, attitude toward AI, and self-efficacy were identified as significant predictors of the latent dimensions of AI anxiety among newly recruited nurses.

**Conclusion:**

Heterogeneity exists in AI anxiety among newly recruited nurses, suggesting that nursing managers should focus on the “high anxiety-technology rejection” group by providing targeted AI training and psychological support to enhance these nurses’ acceptance and application of AI technology.

## 1 Introduction

AI is an emerging scientific and technological field that applies computers to simulate human intelligence and problem-solving abilities (1). With the rapid development of AI technology in the healthcare sector, smart nursing devices and AI-assisted diagnosis and treatment systems have gradually been integrated into clinical nursing practice, presenting new opportunities to enhance nursing efficiency and quality (2). Research indicates that AI will transform the competency requirements in the nursing field (3). However, as the new generation of the nursing workforce, newly recruited nurses often lack clinical experience and are still developing their professional skills. Faced with the rapid iteration and clinical adoption of AI technology, they are prone to anxiety regarding technology learning, job displacement, and career development (4). AI anxiety—defined as the negative psychological reactions such as tension, worry, and resistance that individuals experience when confronted with the application of AI technology—not only reduces nurses’ willingness to adopt and actively use intelligent technologies but may also impact clinical efficiency (5), nursing quality, and even occupational mental health, thereby hindering the development and promotion of smart nursing systems (6, 7).

Currently, research on AI anxiety is predominantly found in fields such as education and management (8, 9), while studies targeting healthcare professionals—particularly newly recruited nurses—are still in their infancy. Furthermore, existing research on AI anxiety among nurses has largely focused on general status surveys, with insufficient attention paid to heterogeneous characteristics within the group; thus, the potential categories and differences in influencing factors among newly recruited nurses with varying levels of anxiety have yet to be revealed. Therefore, this study aims to identify latent categories of AI-related anxiety among newly recruited nurses through latent profile analysis, explore their distribution characteristics and associated influencing factors, and provide a scientific basis for developing targeted intervention strategies and enhancing newly recruited nurses’ adaptability to AI technology.

## 2 Subjects and methods

### 2.1 Study population

Convenience sampling was used to select newly recruited nurses from four Grade A tertiary hospitals in Liaoning, Shandong, and Jilin, China, as the study subjects. Inclusion criteria: ① Possession of a valid nursing license and completion of hospital registration; ② Nurses who have been working in a hospital for no more than two years since graduating from a medical college; ③ Nurses who are aware of and voluntarily participate in this study, having provided informed consent. Exclusion criteria: ① Those who were absent from work for an extended period during the survey period due to sick leave, personal leave, or off-site training; ② Nurses who had not yet been formally hired, interns, nursing aides, and other temporary nursing staff.

Based on the recommendations of Nylund-Gibson et al. (10), a minimum sample size of 300 participants is recommended for latent profile analysis (LPA) to ensure adequate identification of smaller latent profiles. Additionally, general guidelines for multivariate analysis suggest that the sample size should be at least five times the number of observed variables (11).

In this study, the number of observed variables includes 7 demographic variables, level of AI Anxiety (21 items), Attitudes toward Artificial Intelligence (15 items), and level of General Self-Efficacy (10 items), resulting in a total of 53 observed variables. Thus, the formula for sample size is *N* = 53*5 = 265, which means that at least 265 subjects are needed for this study. Also, the sample size should be further expanded considering the sample loss rate of 20%. Therefore, the minimum sample size required is *N* = 265* (1 + 20%) ≈ 318. The final sample comprised 715 participants, which exceeded the required threshold and was therefore deemed sufficient for the planned analyses.

#### 2.1.1 Ethics approval and informed consent

This study complies with the ethical standards of the Declaration of Helsinki. All participants were provided with an informed consent form prior to the commencement of the study. Participants were informed that they could withdraw from the study at any time. All information is kept strictly confidential and is used solely for the purposes of this study. This study has been approved by the ethics committee (JZMULL2026333).

### 2.2 Survey instruments

#### 2.2.1 Demographic questionnaire

Designed by the research team, this questionnaire included questions on age, gender, educational level, department, income level, whether the respondent had received AI training, and their proficiency in AI technology.

#### 2.2.2 Artificial intelligence anxiety scale

Developed by Wang et al. (12), this scale consists of 21 items across four dimensions: Individual Learning (8 items), Job Replacement (6 items), Social-Technological Blind Spots (4 items), and AI Deployment (3 items). It employs a 5-point Likert scale, where 1 indicates “strongly disagree” and 5 indicates “strongly agree.” The total score ranges from 21 to 105 points, with higher scores indicating greater levels of anxiety. The scale’s Cronbach’s *α* is 0.964. In this study, the Cronbach’s α of this scale is 0.909.

#### 2.2.3 Attitude scale towards the use of artificial intelligence technologies in nursing (ASUAITIN)

This scale was developed by Yilmaz et al. (13) and serves as a reliable tool for assessing clinical nurses’ attitudes toward the use of artificial intelligence in nursing practice. The scale consists of 15 items organized into two dimensions: positive attitudes toward the use of artificial intelligence technology in nursing practice (9 items) and negative attitudes toward the use of artificial intelligence technology in nursing practice (6 items). Each item is rated using a 5-point Likert scale (1 = strongly disagree, 5 = strongly agree), with higher scores indicating a more positive attitude. The Cronbach’s *α* coefficient for the total scale was 0.910, with a Cronbach’s α of 0.933 for the negative attitude dimension and 0.917 for the positive attitude dimension. Hu et al. adapted the scale into Chinese based on the current status of AI technology application in nursing practice in China; after cross-cultural adaptation, the Chinese version of the scale was finalized (14). The Chinese version of the scale demonstrates good internal consistency, with a Cronbach’s *α* of 0.785 for the total scale, 0.920 for the negative attitude dimension, and 0.948 for the positive attitude dimension. In this study, the Cronbach’s *α* of this scale is 0.712, 0.704 for the negative attitude dimension, and 0.712 for the positive attitude dimension.

#### 2.2.4 General self-efficacy scale (GSES)

Developed by Schwarzer and Born (15) and revised by Caikang (16). It consists of 10 items and is a unidimensional scale. A 4-point scoring system is used, with 1 point for “completely incorrect” and 4 points for “completely correct.” The total score ranges from 10 to 40, with higher scores indicating higher self-efficacy. The Cronbach’s *α* coefficient for this scale is 0.870. In this study, the Cronbach’s α of this scale is 0.793.

### 2.3 Data collection method

Surveyors who had undergone standardized training, after consulting with medical staff, administered the questionnaire to newly recruited nurses who met the inclusion and exclusion criteria using a standardized set of instructions. After obtaining consent, the questionnaires were distributed, collected, and checked on the spot; any errors or omissions were corrected immediately. A total of 740 questionnaires were distributed in this study, and 715 valid questionnaires were recovered, resulting in a valid response rate of 96.62%.

### 2.4 Statistical methods

We used Mplus 8.3 software to conduct latent profile analysis on the mean scores of various dimensions of AI-related anxiety among newly recruited nurses, gradually increasing the number of categories in the model until the model fit indices reached their optimal levels. Model fit evaluation metrics included the Akaike Information Criterion (AIC), the Bayesian Information Criterion (BIC), and the sample size-adjusted Bayesian Information Criterion (aBIC). Lower values for these three metrics indicate better model fit and higher performance; the entropy index is used to evaluate classification accuracy, with values ranging from 0 to 1. When the entropy index is greater than 0.80, it indicates that over 90% of cases are classified correctly, and the closer the value is to 1, the more accurate the classification; The Likelihood Ratio Test (LMR) and the Bootstrapped Likelihood Ratio Test (BLRT) were used to compare differences in model fit. When the *p*-values for both tests reached the significance level, this indicates that the k-class model is significantly superior to the k-1-class model. BLRT bootstrap resamples: 500 bootstrap draws, random start configuration: STARTS = 1,000 200 (1,000 initial random starting values to avoid local maxima; 200 final optimization iterations). Fixed random seed for all estimation runs: SEED = 12,345.

Data analysis was performed using SPSS 26.0 software. Chi-square tests, Fisher’s exact test, and the Kruskal-Wallis H test were used to compare differences in the distribution of relevant variables across latent categories, while multiclass logistic regression was used to analyze the influencing factors of different latent categories. The significance level was set at *α* = 0.05.

## 3 Results

### 3.1 Participant characteristics

A total of 740 questionnaires were distributed in this study. After screening, 25 were excluded, resulting in 715 valid questionnaires, with a valid response rate of 96.62%. Reasons for exclusion included: incomplete data (*n* = 11) and participation in other related studies (*n* = 14). Other basic information is shown in Table 1.

**Table 1:** Demographic characteristics of newly recruited nurses.

Variable	Category	Number of cases
Age	≤22	282 (39.4)
	23–27	272 (38.0)
	≥28	161 (22.5)
Gender	Female	563 (78.7)
	Male	152 (21.3)
Education Level	College	129 (18.0)
	Bachelor’s degree	419 (58.6)
	Graduate	167 (23.4)
Department	Internal medicine	246 (34.4)
	Surgery	236 (33.0)
	ICU	116 (16.2)
	Emergency	71 (9.9)
	Operating room	46 (6.4)
	Income = Expenditure	234 (32.7)
	Income < expenditures	276 (38.6)
Have you received training in artificial intelligence technology?	Yes	377 (52.7)
	No	338 (47.3)
Level of Proficiency in Artificial Intelligence Technology	Not proficient	201 (28.1)
	Average	311 (43.5)
	Good	203 (28.4)
Attitudes toward AI		41 (32, 45)
Self-efficacy		28 (23, 31)

The results of normality test indicated that the scores of artificial intelligence anxiety, ASUAITIN and GSES among newly recruited nurses did not conform to the normal distribution (*p*<0.05).

The results showed that the AI anxiety score among newly recruited nurses was 45.00 (37.00, 57.00). Score for the Individual Learning dimension is 16.00 (12.00, 22.00), Score for the Job Replacement dimension is 12.00 (9.00, 15.00), Score for the Social-Technological Blind Spots dimension is 8.00 (6.00, 10.00), Score for the AI Deployment dimension is 9.00 (7.00, 10.00).

The ASUAITIN scale score is 41.00 (32.00, 45.00), among which the negative dimension score is 17.00 (13.00, 19.00), score of positive dimension is 24.00 (20.00, 27.00).

The score of the GSES scale is 28.00 (23.00, 31.00).

### 3.2 Latent profile analysis of AI anxiety among newly recruited nurses

**Figure 1:**
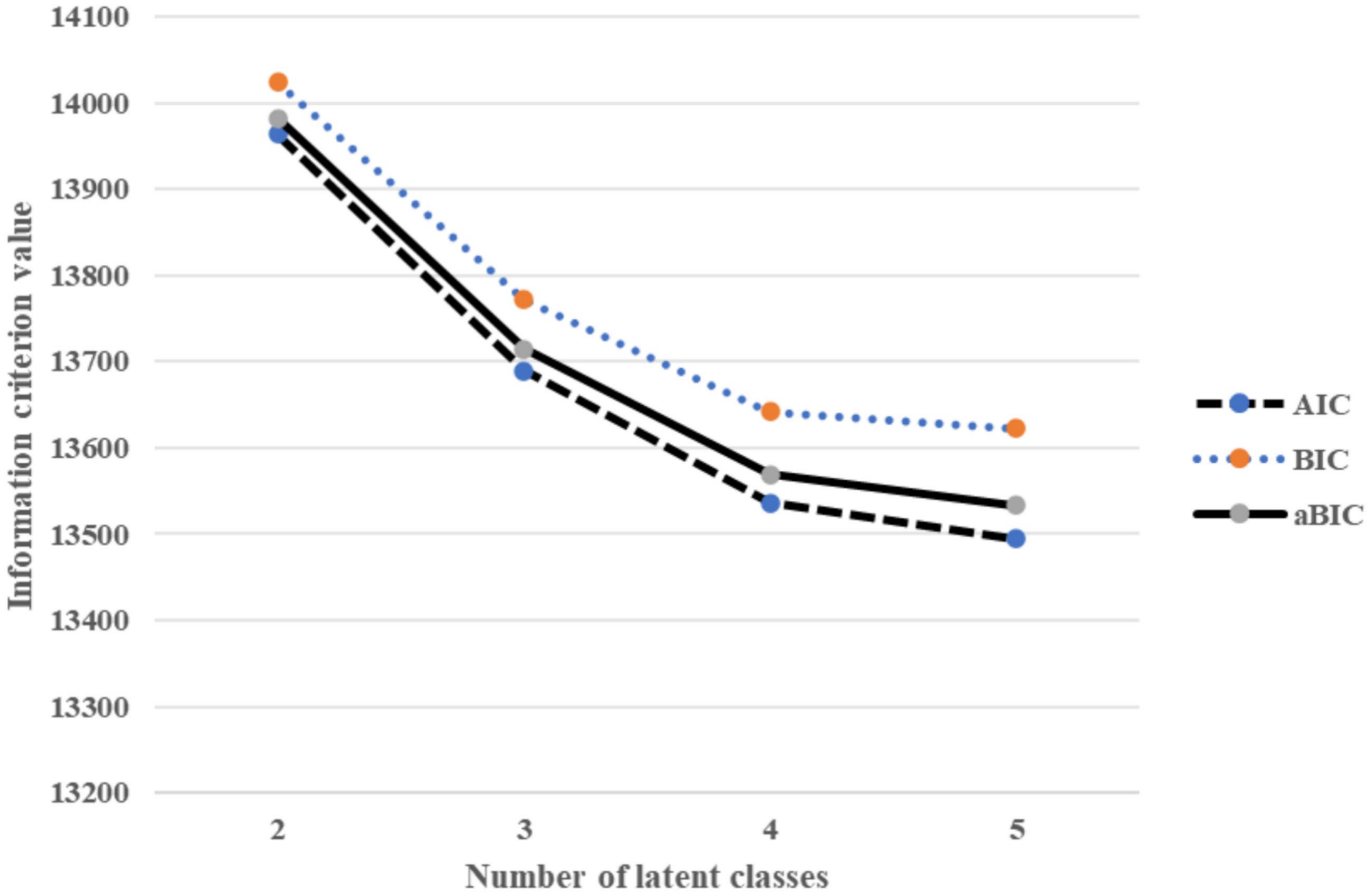
Scree plot of fit indices for latent profile models.

The model fit indices for this study are presented in Table 2. As the number of extracted latent profiles increased from 1 to 3, AIC/BIC and aBIC both showed a significant downward trend, with statistically significant results in the LMR and BLRT tests (all *p* < 0.001); the entropy index increased and approached 1. When the number of latent profiles increased from 3 to 4, the entropy index decreased and the proportion of categories was smaller. Despite continuous decreases in AIC, BIC and aBIC from the 3-class to 5-class models, the 5-class model showed a non-significant LMR test result, and both 4- and 5-class models had lower entropy values indicating poorer classification precision. Although a 9.8% subgroup in the 5-class model meets basic numerical estimation requirements, it lacks practical value for hospital nursing management. A scree-type plot of information criteria are shown in the Figure 1, scree plot shows that the decline rate of the three information criteria slows sharply after the 3-class point (elbow point), visually proving that the incremental fitting gain of 4/5-class models is negligible. The 3-class structure achieves sufficient differentiation with fewer latent groups, complying with the parsimony principle.

**Table 2:** Results of model fit parameters for different latent profile models (*n* = 715).

Model	AIC	BIC	aBIC	Entropy	LMR (*p*)	BLRT (*p*)	Class probability
2	13,964.029	14023.468	13,982.190	0.895	<0.001	<0.001	0.568/0.432
3	13,689.820	13,772.121	13,714.966	0.863	<0.001	<0.001	0.476/0.375/0.150
4	13,536.714	13,641.877	13,568.846	0.825	<0.001	<0.001	0.369/0.302/0.194/0.134
5	13,494.570	13,622.594	13,533.687	0.807	0.156	<0.001	0.364/0.214/0.098/0.130/0.194

**Figure 2:**
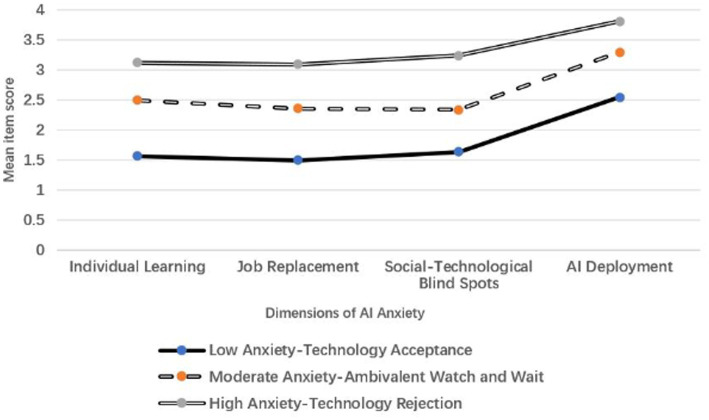
Distribution characteristics of AI anxiety among newly employed nurses.

After a comprehensive comparison of the fit indices of each model, Model 3 was identified as the best-fitting model. Based on Model 3, the scores of each profile across the dimensions of the Artificial Intelligence Anxiety Scale are shown in the Figure 2.

Among them, the Category 1 comprised 340 cases (47.6%), representing the largest demographic in this study. This group scored the lowest across all dimensions of AI anxiety, demonstrated high acceptance of AI technology, and exhibited relatively mild professional concerns and reluctance toward learning. This indicates that they have a more comprehensive understanding of AI technology, a high level of acceptance of AI-assisted nursing work, fewer concerns about AI replacing their jobs, and do not perceive any significant application gaps in AI technology. Nurses in this category may be more willing to learn new technologies and serve as a driving force for promoting AI applications in the nursing field. They are designated as the “Low Anxiety–Technology Acceptance” type.

Category 2 comprises 268 cases (37.5%). This group has moderate anxiety scores across all dimensions, holding conflicting views on the application value and potential risks of AI technology. Acceptance and concern coexist, and they remain in a state of overall wait-and-see. The stable overall trend indicates that their attitude toward AI is contradictory: while they recognize the potential of AI technology in nursing work, they also harbor certain concerns about the potential professional impact and technical risks it may bring. These nurses represent the “middle force” within the nursing community, and are designated as the “Moderate Anxiety-Ambivalent Watch and Wait” Type. Through targeted public education and training, this category can as much as possible to the “low anxiety-technology acceptance” type.

Category 3 comprises 107 cases (15.0%). This group scored significantly higher than the other two categories across all dimensions of AI anxiety, particularly in job displacement and individual learning. They exhibit strong resistance to AI technology and a sense of occupational insecurity, making them a high-risk group for AI anxiety. These nurses constitute a high-risk group for AI anxiety and may exhibit cognitive biases regarding new technologies and low information literacy. Designated as “high anxiety-technology rejection” type, they are the primary focus for subsequent interventions.

### 3.3 Univariate analysis of potential dimensions of AI anxiety among newly recruited nurses

The analysis results indicate that the three latent categories of AI anxiety among newly recruited nurses exhibit statistically significant differences in terms of age, educational level, income level, whether they have received AI training, level of AI proficiency, attitude toward AI, and self-efficacy (all *p* < 0.05), as shown in Table 3.

**Table 3:** Univariate analysis of latent dimensions of AI anxiety among newly recruited nurses (*n* = 715).

Item	Frequency	Class 1^a^ (*n* = 340)	Class 2^b^ (*n* = 268)	class3^c^ (*n* = 107)	χ^2^/HC	*p*
Age						
≤22	282 (39.4)	109 (38.7)	118 (41.8)	55 (19.5)	18.059	<0.001
23–27	272 (38.0)	147 (54.0)	89 (32.7)	36 (13.2)		
≥28	161 (22.5)	84 (52.2)	61 (37.9)	16 (9.9)		
Educational attainment					60.044	<0.001
College	129 (18.0)	39 (30.2)	49 (38.0)	41 (31.8)		
Bachelor’s degree	419 (58.6)	191 (45.6)	171 (40.8)	57 (13.6)		
Graduate	167 (23.4)	110 (65.9)	48 (28.7)	9 (5.4)		
Income level					194.941	<0.001
Income > Expenditure	205 (28.7)	176 (85.9)	21 (10.2)	8 (3.9)		
Income = Expenditure	234 (32.7)	103 (44.0)	98 (41.9)	33 (14.1)		
Income < Expenditure	276 (38.6)	61 (22.1)	149 (54.0)	66 (23.9)		
Have you received training in artificial intelligence technology?					67.796	<0.001
Yes	377 (52.7)	234 (62.1)	105 (27.9)	38 (10.1)		
No	338 (47.3)	106 (31.4)	163 (48.2)	69 (20.4)		
Level of proficiency in artificial intelligence technology					53.249	<0.001
Unskilled	201 (28.1)	68 (33.8)	81 (40.3)	52 (25.9)		
Average	311 (43.5)	141 (45.3)	134 (43.1)	36 (11.6)		
Good	203 (28.4)	131 (64.5)	53 (26.1)	19 (9.4)		
Attitudes toward Artificial Intelligence		45 (41, 48)	37 (32, 43)	28 (27, 30)	320.983	<0.001
Self-efficacy		31 (29, 32)	24 (21, 27)	20 (18, 21)	401.658	<0.001

a Low anxiety-technology acceptance.

b Moderate anxiety-ambivalent watch and wait.

c High anxiety-technology rejection.

### 3.4 Multivariate analysis of potential dimensions of AI anxiety among newly recruited nurses

Using the three latent categories of AI anxiety among newly recruited nurses as the dependent variables, variables showing statistically significant differences in the univariate analysis were included in the multivariate logistic regression analysis.

Multinomial logistic regression reference specifications: Outcome reference group = High Anxiety-Technology Rejection (Class 3). Predictor reference groups: Graduate degree (education level), income > expenditures (income level), received AI training (AI training), good AI proficiency (technical skill). The results are shown in Table 4.

**Table 4:** Multivariate logistic regression analysis of latent dimensions of AI anxiety among newly recruited nurses (*n* = 715).

Variable	β	SE	Wald χ^2^	*P*	OR	95% CI
Class 1^a^ vs. Class 3^c^						
Constant	−26.578	2.168	150.251	<0.001		
Education level (college)	−1.505	0.696	4.680	0.031	0.222	0.057–0.868
Income level (income > expenditures)	1.861	0.722	6.651	0.010	6.430	1.563–26.453
Have you received training in artificial intelligence technology? (yes)	0.958	0.411	5.434	0.020	2.606	1.165–5.831
Level of proficiency in AI technology (unskilled)	−1.190	0.559	4.535	0.033	0.304	0.102–0.910
AI attitude	0.359	0.041	75.248	<0.001	1.432	1.321–1.553
Self-efficacy	0.611	0.062	97.745	<0.001	1.843	1.632–2.080
Class 2^b^ vs. Class 3						
Constant	−11.666	1.570	55.183	<0.001		
Education level (college)	−1.208	0.546	4.898	0.027	0.299	0.102–0.871
AI attitude	0.226	0.036	40.300	<0.001	1.254	1.169–1.344
Self-efficacy	0.284	0.050	31.912	<0.001	1.328	1.204–1.466

a Low anxiety-technology acceptance.

b Moderate anxiety-ambivalent watch and wait.

c High anxiety-technology rejection.

The findings of this study suggest that educational level is an important factor associated with the latent profiles of artificial intelligence anxiety among newly recruited nurses. Using the “high anxiety-technology rejecting” type as the reference group, nurses with an associate degree were classified as low anxiety-technology acceptance type (OR = 0.222, 95% CI: 0.057–0.868, *p* = 0.031) and the moderate anxiety-ambivalent watch and wait type (OR = 0.299, 95% CI: 0.102–0.871, *p* = 0.027); income level had a significant impact only on the formation of the low anxiety-technology acceptance type; nurses with income exceeding expenditure had a significantly higher probability of belonging to this group (OR = 6.430, 95% CI: 1.563–26.453, *p* = 0.010); whether nurses had received AI technology training was an independent positive factor influencing the low anxiety-technology acceptance type, with nurses who had received such training showing a significantly higher probability of belonging to this group (OR = 2.606, 95% CI: 1.165–5.831, *p* = 0.020); AI proficiency is a key factor influencing the formation of the low anxiety-technology acceptance type; nurses with low AI proficiency had a significantly lower probability of belonging to this type (OR = 0.304, 95% CI: 0.102–0.910, *p* = 0.033).

Attitude toward AI is a stable, positive factor influencing the latent categories of anxiety among newly recruited nurses. Nurses with a more positive attitude toward AI had a significantly higher probability of being classified as the low anxiety-technology acceptance type (OR = 1.432, 95% CI: 1.321–1.553, *p* < 0.001) and the moderate anxiety-ambivalent watch and Wait type (OR = 1.254, 95% CI: 1.169–1.344, *p* < 0.001).

Self-efficacy is a core important factor influencing the latent categories of anxiety among newly recruited nurses; nurses with higher self-efficacy were significantly more likely to be classified as low anxiety-technology acceptance (OR = 1.843, 95% CI: 1.632–2.080, *p* < 0.001) and the moderate anxiety-ambivalent watch and wait type (OR = 1.328, 95% CI: 1.204–1.466, *p* < 0.001).

## 4 Discussion

### 4.1 Current status and potential profile characteristics of AI anxiety among newly recruited nurses

The results showed that the AI anxiety score among newly recruited nurses was 45.00 (37.00, 57.00), this indicates a relatively high level of anxiety. This is consistent with 43.05 ± 13.29 in the existing research findings (17). Studies from Turkey show that nurses across various departments experience digital anxiety of varying degrees (18, 19), confirming that technology-related apprehension toward clinical AI is a pervasive workforce issue across Asian and Mediterranean healthcare systems. This study classified AI anxiety among newly recruited nurses into three potential subtypes: low anxiety-technology acceptance, moderate anxiety-ambivalent watch and wait, and high anxiety-technology rejection.

The low anxiety-technology acceptance type accounted for 47.6%. Nurses in this category exhibit high acceptance of AI technology, low anxiety levels, and a strong willingness to learn, making them a key reserve force for the intelligent transformation of nursing. They perceive high perceived usefulness of AI nursing tools, possess sufficient digital literacy to reduce perceived ease-of-use barriers, and hold robust general self-efficacy that buffers technological stress. This is consistent with the findings of a study on medical students’ attitudes toward artificial intelligence (20), who similarly exhibit low AI fear when equipped with formal digital training and positive perceived utility beliefs. Nursing managers may tap this cohort’s positivity and adaptability by integrating them into ward intelligent nursing teams. Experience sharing and skill demonstrations build departmental acceptance of AI nursing tools. Meanwhile, tiered AI training, academic exchanges and career pathways boost their technical proficiency and professional belonging, translating individual strengths into team resilience and cultivating a constructive ward culture.

The moderate anxiety-ambivalent watch and wait type accounts for 37.5%. Nurses in this category exhibit moderate anxiety levels, wavering attitudes, and the highest level of adaptability. Their coexisting recognition of AI’s clinical benefits and fear of occupational displacement aligns with paediatric nurses’ mixed attitudes observed in Türkiye (18). The conflicting mindset of this group often stems from incomplete understanding of AI technology, uncertainty about career prospects, and insufficient skill reserves. Nursing managers shall launch targeted measures including regular AI nursing lectures. Real clinical cases can clarify AI’s auxiliary role and dispel the myth that AI replaces nurses. Basic skill training eases learning barriers and anxiety to foster AI operation competence. Meanwhile, career planning shall be embedded in new nurse orientation to illustrate nursing’s prospects amid intelligent healthcare.

The high anxiety-technology rejection type (15.0%), which represents the smallest proportion but poses the highest risk, exhibits pronounced anxiety across all dimensions. Mechanistically, this group operates under a deficit-cycle feedback loop: low AI proficiency generates repeated operational failure, which erodes self-efficacy, amplifies catastrophic thinking about technological displacement, and reinforces active resistance to AI training—consistent with the vicious cycle model proposed in physician AI anxiety research (20). The primary focus of intervention for this group should be on risk warning, targeted intervention, psychological counseling, and enhanced support. Nursing managers shall set up early screening and dynamic monitoring for anxious nurses during pre-job training. Deliver priority one-on-one cognitive-behavioral interventions to resolve AI-related anxiety and negative biases. Offer tailored technical training and regular psychological counseling to ease stress, burnout and turnover risks, lower tech anxiety, stabilize mental status and retain new nurses.

### 4.2 Analysis of factors influencing potential categories of AI-related anxiety among newly recruited nurses

#### 4.2.1 Educational level

Educational attainment affects various latent AI-related anxiety profiles among newly recruited nurses. The higher the educational level, the less likely nurses are to develop negative coping patterns characterized by high anxiety and strong rejection. This finding is consistent with the study by Lui et al. (21), its research shows that, Graduate and bachelor’s nurses receive systematic information literacy and critical thinking training during university, which decouples AI cognition from simplistic “technology replaces humans” narratives. At the same time, nurses with higher educational attainment possess stronger learning abilities and environmental adaptability. When facing the intelligent transformation of nursing, they are more likely to alleviate technology-related anxiety through active learning and independent exploration, thereby developing a rational and accepting attitude (17). This suggests that in nursing talent development, it is necessary to further refine intelligent skills curricula for nurses at different educational levels (22), with a particular focus on strengthening information literacy education for nurses with associate degrees. This will help narrow the technological adaptation gap caused by educational background and reduce the risk of high-anxiety, rejection-oriented attitudes.

#### 4.2.2 Income level

Income level has a significant impact only on the formation of the low-anxiety, technology-accepting type; nurses with stable financial circumstances are more likely to develop positive patterns of technological adaptation. Economic pressure is a significant social factor affecting an individual’s mental health and occupational adaptation (23). Newly recruited nurses are at the early stages of their careers, and their income levels are generally low. Some nurses face the dilemma of an imbalance between income and expenditure; additional financial burdens amplify their concerns about the uncertainty of career development and intensify their sense of occupational crisis regarding “AI replacing their jobs” (24), and this may further raise the possibility that they will fall into the group with high anxiety-technology rejection type. Conversely, nurses with balanced budgets and minimal financial pressure can more calmly address the challenges posed by career transitions and skill acquisition, reducing the negative impact of economic factors on their attitudes toward technology adaptation (25). This indicates managers should care for nurses’ finances, ease economic stress via better benefits and living support to help them adapt to smart nursing.

#### 4.2.3 AI technology training

Whether or not one has received AI technology training is an independent positive factor influencing the low-anxiety-high-technology-acceptance model. Multiple studies have confirmed that: Systematic AI training is a key means of alleviating AI-related anxiety among newly recruited nurses and promoting their active acceptance (26); one of the primary sources of AI anxiety is a “sense of technological unfamiliarity” and “fear of operation,” and systematic training can effectively bridge this information gap and address competency gaps (6). Trained nurses not only gain a systematic understanding of AI’s application scenarios, operational procedures, and risk management in clinical nursing—thereby correcting the misconception that “AI will replace nurses”—but also enhance their technical proficiency through hands-on practice. This reduces their apprehension toward intelligent systems and strengthens their sense of control over the technology (27). This finding also aligns with the consensus in nursing education: intelligent skills training during both pre-service and in-service phases is the most direct and effective way to enhance nurses’ technical adaptability and alleviate technology-related anxiety (28).

#### 4.2.4 Level of AI proficiency

The level of AI proficiency is a key factor influencing the development of a low-anxiety, technology-accepting mindset, while insufficient technical proficiency is a major risk factor driving nurses toward high anxiety and strong resistance. Studies have shown that, the level of AI proficiency determines nurses’ experience and sense of competence when applying AI systems in clinical practice (29). Nurses who are not proficient in AI technology are prone to operational errors and inefficiency when using nursing information systems and intelligent assistive devices. Repeated feelings of frustration further exacerbate their resistance to and anxiety about the technology, creating a vicious cycle of “unable to use → afraid to use → even more resistant” (30); In contrast, nurses proficient in AI technology can smoothly utilize AI tools to complete nursing tasks, tangibly experiencing the convenience and efficiency gains brought by the technology, which in turn fosters a positive attitude of acceptance (31). Managers should prioritize practical AI training over pure theory, add clinical drills and individualized tutoring for less skilled nurses to boost AI proficiency and end skill-anxiety-rejection cycles (32).

#### 4.2.5 Attitudes toward artificial intelligence

Attitudes toward AI are a stable, positive factor influencing the underlying categories of anxiety among newly recruited nurses; a positive attitude toward AI technology serves as a crucial psychological foundation for nurses to develop rational adaptation patterns. This conclusion is consistent with the findings of a study on nurses in Turkey, the findings suggest that lower levels of anxiety related to learning and AI configuration are associated with more positive attitudes toward AI. Attitude is a key predictor of individual behavior and emotional responses (33). Nurses with a positive attitude toward AI are more inclined to proactively seek out technology-related information and focus on its application value in clinical nursing, rather than overemphasizing potential risks (34); this positive cognitive orientation helps nurses embrace the intelligent transformation of nursing with a more open mindset, thereby reducing the emergence of negative emotions and resistant behaviors (35). Conversely, nurses with negative attitudes toward AI are easily influenced by negative narratives such as the “technological replacement theory,” forming irrational stereotypes that further exacerbate anxiety and rejection (36). Interventions including publicity, case sharing and peer communication help new nurses form rational views of AI, fix biased perceptions and reduce tech anxiety cognitively.

#### 4.2.6 Self-efficacy

Self-efficacy is an important influencing factors influencing the underlying categories of anxiety among newly recruited nurses; a high level of self-efficacy serves as a key psychological resource for alleviating AI-related anxiety and promoting positive adaptation. This is consistent with the conclusions of existing studies, nurses with high self-efficacy have greater confidence in their ability to learn new technologies and adapt to professional transitions. When faced with changes brought about by AI, they are able to respond proactively with a positive mindset, reducing anxiety and feelings of overwhelm (36); conversely, nurses with low self-efficacy are prone to doubting their own abilities, overemphasizing the difficulty of learning and applying technology, and thereby exacerbating anxiety and resistance. Enhancing self-efficacy is an effective approach to alleviating occupational stress and technology-related anxiety among nurses (37). Managers may enhance nurses’ self-efficacy via positive feedback, success sharing and staged goals to build confidence and resilience amid smart nursing reform.

## 5 Conclusion

This study identified significant subgroup heterogeneity in AI anxiety among newly recruited nurses via latent profile analysis. Unlike previous studies that only reported overall average levels of nurses’ AI anxiety, this three-class classification reveals divergent psychological adaptation patterns toward intelligent nursing technology within the new nurse workforce, providing a targeted grouping framework for clinical nursing management.

Practically, this three-subgroup structure enables a shift from universal standardized training to stratified precise intervention. Nursing administrators should prioritize targeted psychological counseling and personalized skill training for the high-anxiety technology rejection subgroup, motivate and empower the low-anxiety technology acceptance subgroup to act as peer demonstrators, and carry out cognitive correction and basic technical training for the ambivalent moderate-anxiety group to promote their positive adaptation to AI nursing tools. Stratified interventions based on this latent profile classification can effectively reduce overall AI anxiety among newly recruited nurses, improve their willingness and ability to apply intelligent nursing technology, and support the stable construction of a nursing talent team adapted to intelligent medical transformation.

## 6 Limitations

This study has several limitations. First, the sampling adopted convenience sampling limited to tertiary Grade A hospitals in northern China, excluding primary care institutions, private hospitals and participants from other geographic regions, which restricts the generalizability of findings. Second, this cross-sectional design cannot establish causal relationships between influencing factors and latent AI anxiety profiles; only correlational associations can be inferred. Third, all outcome and predictor data were collected via self-reported questionnaires, which carries an inherent risk of social desirability bias. Newly recruited nurses may underreport their AI anxiety and overstate positive technology attitudes to demonstrate professional adaptiveness, potentially biasing the classification distribution of latent profiles. Finally, several theoretically relevant confounding variables were not measured in the present survey, including quantitative departmental workload disparities (e.g., patient care burden in ICU versus internal medicine), monthly frequency of night shifts, and prior exposure to AI/informatics training during nursing school. These unmeasured factors may independently predict AI anxiety and could confound the observed associations in our logistic regression models. Future research could broaden the range of variables to incorporate more relevant factors for more in-depth analysis.

## 7 Relevance to clinical practice

This study enriches the research achievements in the interdisciplinary field of smart nursing and nursing psychology. It can provide scientific support for pre-job training of newly recruited nurses, the promotion of AI technology, the maintenance of occupational mental health and the stability of nursing teams, and possesses important theoretical and practical significance for advancing the intelligent transformation of clinical practice.

Abandon the one-size-fits-all training model, propose hierarchical and categorized precise intervention strategies, provide nursing managers with implementable solutions for early screening, risk early warning and targeted support, and advance the transformation of nursing management from experience-driven to data-driven.

This study addresses the practical dilemmas and psychological needs of nursing staff amid the intelligent transformation of healthcare, focusing on both technological application and humanistic care. It holds long-term significance for advancing the high-quality development of the nursing industry, safeguarding the mental health of medical staff, and improving the overall efficiency of medical services.

## Data Availability

The raw data supporting the conclusions of this article will be made available by the authors, without undue reservation.
